# Congenital pouch colon: Increasing association with low anorectal anomalies

**DOI:** 10.4103/0971-9261.59606

**Published:** 2009

**Authors:** Arunachalam Pavai, Suma D. Pillai, S. Shanthakumari, Cenita J. Sam, M. Shylaja, R. Sabarivinoth

**Affiliations:** Departments of Paediatric Surgery, Pathology, P.S.G Institute of Medical Sciences and Research and Hospitals, Peelamedu, Coimbatore 641 004, India

**Keywords:** Congenital pouch colon, anorectal malformation, constipation, mega rectum

## Abstract

Three cases of type IV congenital pouch colon associated with low anorectal anomaly are reported here. Pouch colon may be a cause of intractable constipation in children operated for low anorectal anomaly. Excellent results can be obtained by exicision of the pouch. The radiological and pathological features of this condition are discussed.

## INTRODUCTION

Congenital pouch colon (CPC) is a condition associated with anorectal malformations (ARM). Ninety percent of CPC are reported from the Indian subcontinent and predominantly from the northern provinces.[1–6] Earlier, type I CPC with high ARM was found to be more common, but over the years incidence of type IV CPC is increasing and there have been isolated reports of CPC with low ARM.[3,7–9] We report three cases of type IV CPC with low ARM, their histopathological findings, and the importance of considering CPC in low ARM when there is persistent constipation.

## CASE REPORTS

### Case 1

**Figure 1:**
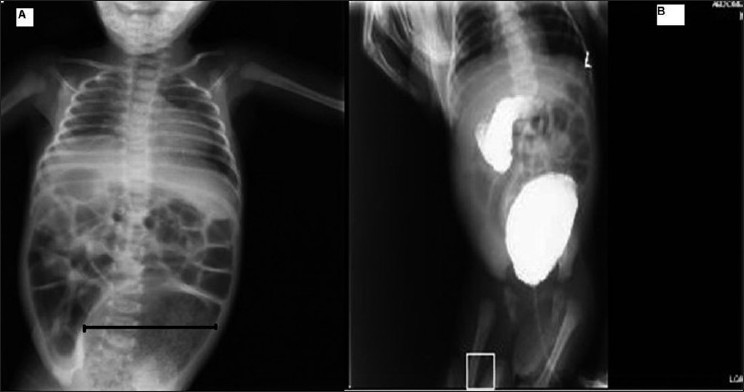
Plain X-ray showing dilated rectosigmoid, gas shadow occupying >50% of abdominal width. (b) Barium enema showing dilated rectosigmoid.

A 20-day-old female neonate presented with severe diarrhea. She was moderately dehydrated with a distended abdomen and was diagnosed to have diarrhea with an anovestibular fistula and associated UTI. The vestibular opening admitted an 8 Fr dilator. Skiagram of the abdomen showed a dilated rectosigmoid (gas shadow occupying >50 % of the width of the abdomen). After stabilization, barium enema showed dilated rectum and sigmoid with abrupt transition to normal proximal colon [Figure 1a,b]. There was history of straining to pass stool prior to the diarrhea. USG abdomen and CT showed an L-shaped kidney with left hydroureteronephrosis. Laparotomy revealed pouch involving the whole of the rectum and proximal sigmoid with abrupt transition to normal descending colon. End colostomy proximal to the pouch was done and a biopsy was sent from the hypertrophied sigmoid segment. Normal ganglion cells were found. After 6 months, excision of pouch and pull through was done. Histopathology of the pouch revealed lymphoid follicles and fibrosis in the mucosa with the presence of normal ganglion cells in between the circular and longitudinal layers of muscularis propria and disarray in the arrangement of the muscle fibers in the muscle coat. Post-operatively the baby is passing stools daily. Cystoscopy had shown left ectopic ureter and baby has undergone ureteric reimplantation.

### Case 2

A male neonate was referred with multiple congenital anomalies. Physical examination revealed absent pinna on the right side, tuft of hair on the back, absent anal opening, and abdominal distension. The child was evaluated for possible associated anomalies and diagnosed to have Goldenhar's syndrome. Prone cross-table lateral X-ray was suggestive of low anorectal anomaly and a moderately dilated rectum, and this was attributed to the obstruction. Anoplasty was done. Renal ultrasound was normal. He had persistent constipation without any anal stenosis. A contrast was done in order to rule out pouch colon as he had a moderately dilated rectum in the newborn period. Contrast X-ray was diagnostic of rectal pouch and hence excision of pouch and pull through was done. Post-operatively the constipation resolved. HPE of the pouch revealed mucosal lymphoid aggregates, muscle hypertrophy, and disarray in certain areas with normal ganglion cells.

### Case 3

**Figure 2:**
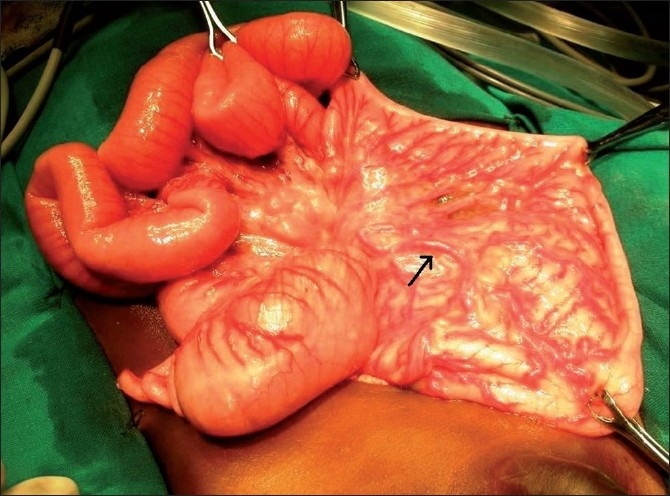
Pouch colon involving the rectosigmoid with abnormal leash of blood vessels supplying it depicted by an arrow.

A 1-year-old male child was referred with a mass in the abdomen with constipation and failure to thrive. He had undergone anoplasty for imperforate anus soon after birth at another hospital and was on laxatives for the constipation. Examination revealed normal external genitalia and a loaded colon. Per rectal examination revealed loaded rectum with hard stools. There was no anal stenosis or stricture. Barium enema revealed dilated sigmoid and rectum with abrupt transition to normal colon. Laparotomy revealed a typical type IV pouch involving the sigmoid and rectum. The pouch was supplied by a leash of vessels arising from inferior mesenteric artery. There were no appendices epiploicae, haustrations, and taenia coli, and there was abrupt transition to normal colon proximally [Figure 2]. Excision of the pouch and endorectal pull through was done. HPE of the pouch revealed lymphoid follicles and fibrosis in the mucosa, submucosal thinning, muscular hypertrophy, and disorganization and normal ganglion cells. Normal stool pattern was noted at 2 years follow-up and he had attained the 95th centile in his growth chart.

## DISCUSSION

CPC is an extremely rare variant of anorectal malformation in which all or part of the colon is replaced by a pouch-like dilatation that communicates distally with the urogenital tract by a large fistula. They are divided into four types.[2]

Type I – Normal colon is absent and the ileum opens directly into the colonic pouch.

Type II – The ileum opens into a short segment of cecum, which then opens into the pouch.

Type III – Presence of a significant length of normal colon between the ileum and the colonic pouch.

Type IV – Presence of near normal colon with only the terminal portion of colon (sigmoid and rectum) converted into a pouch.

Over the years, there has been a trend of reducing severity of pouch colon, that is, from Type I to Type IV.[7] Pathak *et al.* in his article reports only one case of type IV CPC out of a total of 56 cases of pouch colon between 1968 and 1984, and the figure rose to 46 out of a total of 81 cases between 1985 and 1999.[9] Other studies also show an increase in the incidence of incomplete pouch colon than complete variety.[10] CPC occurred in up to 13% of all malformations and 27% of high ARM.

We treated three cases of type IV CPC (two males and one female) within a period of 2 years. All three cases were associated with low ARM. This supports the literature of increasing incidence of type IV CPC. In a study by Chadha *et al.,* 3/10 cases of CPC were type IV and found to be associated with colovestibular fistula.[3] Histology of CPC[5,6,11,12] described in earlier studies revealed normal number of ganglion cells though a few have reported reduced number of ganglion cells. The most salient feature is the disorganization of the muscle coat in an arborizing manner, which is possibly responsible for the absence of normal peristalsis. Other features include nerve bundle hypertrophy, congestion of mucosa, and focal hemorrhages.

Constipation is commonly associated with low ARM.[13,14] Mega rectum after correction of anorectal anomaly is characterized by dilation of the rectum and may be associated with anal stenosis. This condition can be corrected by anal dilatation, re-do anoplasty, and functional toilet training. It is important to differentiate mega rectum from pouch colon when the patient presents at a later date with constipation after anoplasty.[15] In pouch colon, there is no anal stenosis and the dilated pouch has typical radiological and histopathological features. Unlike mega rectum, which is a simple dilatation of the colon because of obstruction, CPC type IV has a defect in peristalsis. Type IV CPC can be corrected surgically by excision of the pouch and pull through. So in any case of persistent constipation following the correction of low ARM without any anal stricture or stenosis, the diagnosis of CPC should be considered, as excision is the definitive management for this condition.
